# Robot-assisted radical cystectomy vs open radical cystectomy in patients with bladder cancer: a systematic review and meta-analysis of randomized controlled trials

**DOI:** 10.1186/s12957-023-03132-4

**Published:** 2023-08-05

**Authors:** Hongquan Liu, Zhongbao Zhou, Huibao Yao, Qiancheng Mao, Yongli Chu, Yuanshan Cui, Jitao Wu

**Affiliations:** 1 Department of Urology, The Affiliated Yantai Yuhuangding Hospital of Qingdao University, NO. 20 East Yuhuangding Road, Yantai, 264000 Shandong China; 2 Department of Urology, Fengtai District, Beijing TianTan Hospital, Capital Medical University. No, 119 South 4Th Ring West Road, Beijing, 100070 China; 3 Department of Scientific Research, The Affiliated Yantai Yuhuangding Hospital of Qingdao University, NO. 20 East Yuhuangding Road, Yantai, 264000 Shandong China

**Keywords:** Bladder cancer, Robot-assisted radical cystectomy, Open radical cystectomy, Randomized controlled trial, Meta-analysis

## Abstract

**Purpose:**

Even though there isn't enough clinical evidence to demonstrate that robot-assisted radical cystectomy (RARC) is preferable to open radical cystectomy (ORC), RARC has become a widely used alternative. We performed the present study of RARC vs ORC with a focus on oncologic, pathological, perioperative, and complication-related outcomes and health-related quality of life (QOL).

**Methods:**

We conducted a literature review up to August 2022. The search included PubMed, EMBASE and Cochrane controlled trials register databases. We classified the studies according to version 2 of the Cochrane risk-of-bias tool for randomized trials (RoB 2). The data was assessed by Review Manager 5.4.0.

**Results:**

8 RCTs comparing 1024 patients were analyzed in our study. RARC was related to lower estimated blood loss (weighted mean difference (WMD): -328.2; 95% CI -463.49—-192.92; *p* < 0.00001), lower blood transfusion rates (OR: 0.45; 95% CI 0.32 – 0.65; *p* < 0.0001) but longer operation time (WMD: 84.21; 95% CI 46.20 -121.72; *p* < 0.0001). And we found no significant difference in terms of positive surgical margins (*P* = 0.97), lymph node yield (*P* = 0.30) and length of stay (*P* = 0.99). Moreover, no significant difference was found between the two groups in terms of survival outcomes, pathological outcomes, postoperative complication outcomes and health-related QOL.

**Conclusion:**

Based on the present evidence, we demonstrated that RARC and ORC have similar cancer control results. RARC is related to less blood loss and lower transfusion rate. We found no difference in postoperative complications and health-related QOL between robotic and open approaches. RARC procedures could be used as an alternate treatment for bladder cancer patients. Additional RCTs with long-term follow-up are needed to validate this observation.

## Introduction

With more than half a million new cases annually, bladder cancer has become one of the ten most prevalent kinds of cancer in the world [1, 2]. Open radical cystectomy (ORC) is still the advised surgical procedure for patients with muscle-invasive bladder cancer and those with very high-risk non-muscle-invasive bladder cancer [3]. The value of ORC, however, has been limited because of the drawbacks of a high morbidity and mortality, such as urinary tract infection, urinary leak, renal failure, ileus and thromboembolic complications. More than 60% of patients receiving ORC undoubtedly have at least one perioperative problem, and 20% have a high-grade complication after their procedure [4, 5]. Radical cystectomy has been performed using minimally invasive surgical techniques over the past two decades [6]. Menon et al. reported the first robot-assisted radical cystectomy (RARC) twenty years ago [7], which has become common technology around the world, especially in many high-volume locations. Initially, a tiny incision was made in the abdominal wall to execute urinary diversion by extracorporeal procedure. The development of intracorporeal urinary diversion (ICUD) has, however, been made possible by technological adjustments made to RARC. [8]. From 2005 to 2016, the use of ICUD grew from 9 to 97% of urinary diversion surgeries [9].

Several studies have indicated that compared with ORC, RARC has fewer perioperative complications, shorter length of stay and lower estimated blood loss [10–12]. Nevertheless, very few studies have revealed the long-term oncological results, and the majority of investigations are retrospective. Randomized controlled trials (RCTs) remain crucial for establishing how RARC and ORC compare to one another. Recently, a sizable quantity of fresh data from RCTs emerged that included long-term oncological outcomes, adding new evidence to the theme [13–16].

To assess the oncologic, pathological, perioperative, postoperative complications outcomes as well as health-related quality of life (QOL), for RCTs comparing RARC with ORC, we carried out this meta-analysis to update the current evidence base.

## Materials and methods

The PRISMA (Preferred Reporting Items for Systematic Reviews and Meta-Analyses) Statement [17] was followed when conducting this study. As no primary personal information will be gathered, no extra ethical approval is necessary.

### Literature search

The research was registered on PROSPERO (CRD42023396105). Two reviewers independently searched MEDLINE (2009 to August 2022), EMBASE (1995 to August 2022), and the Cochrane Controlled Trials Register databases for appropriate RCTs comparing RARC with ORC. Moreover, references from retrieved studies were searched. The following keywords were applied, such as “bladder cancer”, “cystectomy”, “robot”, “robotic”, “da Vinci”, “ORC”, “RARC”, and “randomized controlled trials”.

### Inclusion criteria and selection of studies

**Fig. 1:**
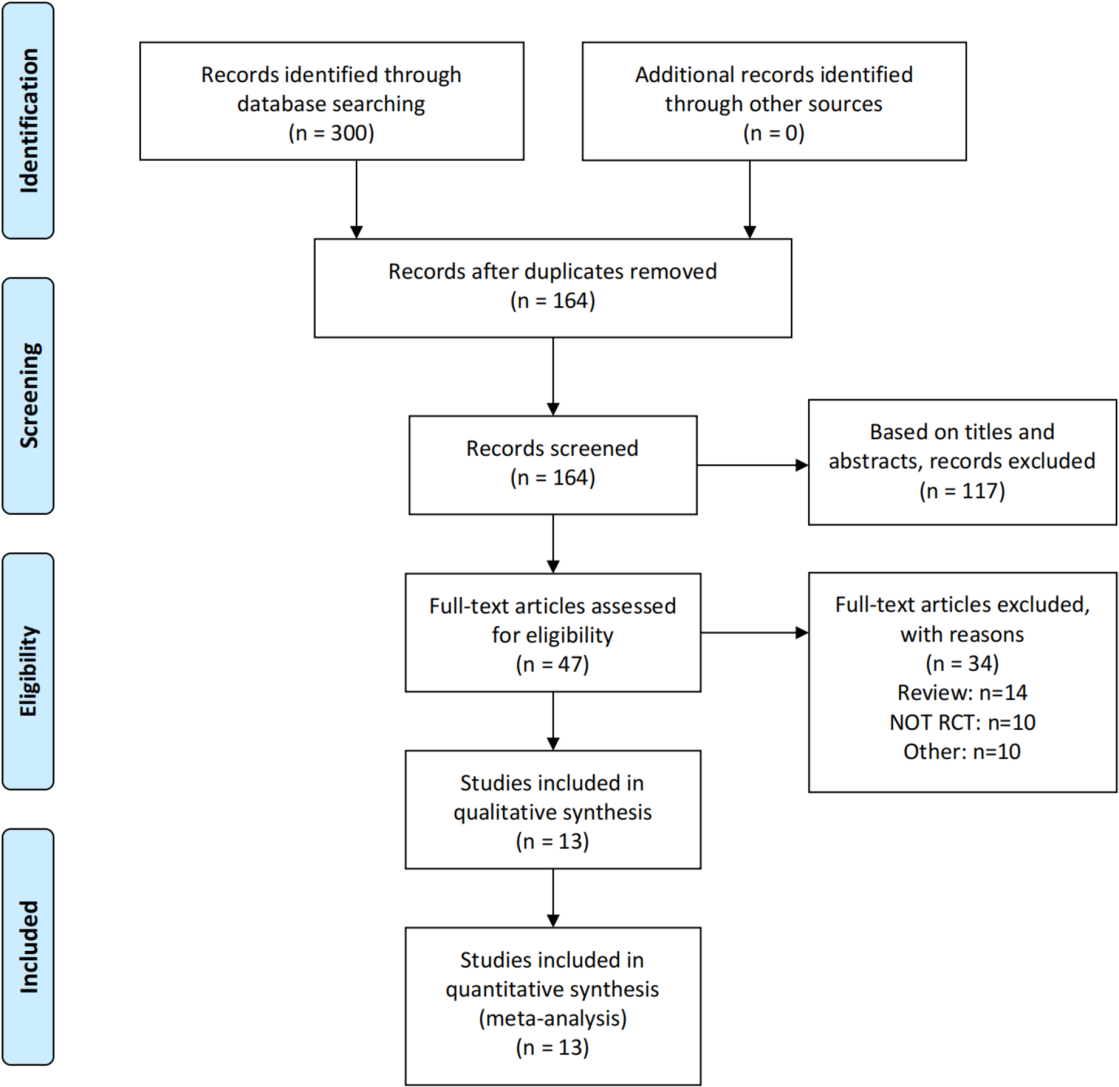
A flow diagram of the study selection. RCT: randomized controlled trial

Studies were included if they conformed to the following criteria: (1) comparison of ORC with RARC; (2) the study provided analyzable data of interest: overall survival (OS), recurrence-free survival (RFS), oncologic efficacy (positive margin status [PSM], lymph node yield, sites of recurrence) and perioperative outcomes (estimated blood loss [EBL], blood transfusion rates, operating room time [ORT], length of stay [LOS]), postoperative complications and health-related QOL assessment; (3) The article's whole text was accessible. We included either the more current or higher-quality patient-cohort article when there were equivalent papers. If the same group performed numerous experiments on a similar set of participants, we included each study. A flowchart of the study selection process is presented in Fig. 1.

### Risk of bias assessment

Using version 2 of the Cochrane risk-of-bias tool for randomized trials (RoB 2) [18], we classified the included studies. These studies were classified into three degrees: low risk of bias, some concerns, or high risk of bias. The writers came to an agreement on certain points where they disagreed.

### Data extraction

We extracted the required data: (1) the first author’s name and the published time of the article; (2) the region of each RCT; (3) study design; (4) treatment and sample size; (5) age, sex, body mass index (BMI) and American Society of Anesthesiologists (ASA) score; (6) tumor stage and urinary diversion type; (7) overall survival (OS) rate, recurrence-free survival (RFS) rate and sites of recurrence; (8) PSM and lymph node yield; (9) blood transfusion rates, ORT, EBL and LOS; (10) the date of postoperative complications and health-related QOL assessment.

### Statistical analysis

This study used Review Manager Version 5.4 (Cochrane Collaboration, London, UK) to evaluate comparable data. Continuous results were estimated using weighted mean difference (WMD) with 95% confidence intervals (CIs). Besides, the odds ratio (OR) was used to estimate dichotomous variables. We used 95% CIs to express all outcomes, and statistical significance was set as *p* < 0.05. The degree of heterogeneity was calculated by Cochrane’s Q test and I^2^ test. If there was significant inconsistency (I^2^ > 50% or *p* < 0.10), we chose a random effect model for meta-analysis; if not, a fixed effect model was applied.

## Results

### Study characteristics

Overall, 300 potentially relevant articles were found by our initial literature search; 136 duplicates were removed. Furthermore, 117 and 34 publications were excluded after evaluating the title/abstract and reading the full-text, respectively. And 13 studies (8 RCTs) [4, 14–16, 19–27] involving 1024 participants were studied in the meta-analysis. The basic features of the included RCTs are listed in Table 1.

**Table 1:** Characteristics of the included studies

Study	Region	Group	Number of patients	Age, years (median or mean)	Sex (M/F)	BMI (mean or median)	ASA score (mean or median)	Tumor stage (T1 or lower) /T2/T3/T4	Diversion type ileal conduit/Neobladder/ continent cutaneous	Rate of neobladder (%)
Catto et al., 2022 [14]	UK	RARC/ORC	161/156	69.3/68.7	128/33; 122/34	NA	NA	71/30/NA/NA 70/34/NA/NA	142/NA/NA; 140/NA/NA	NA
Vejlgaard et al., 2022 [20]	Denmark	RARC/ORC	25/25	70/67	18/7; 20/5	27.3/26.9	2.12/2	9/13/3/NA 6/15/4/NA	25/0/0; 25/0/0	0/0
Maibom et al., 2022 [16]	Denmark	RARC/ORC	25/25	70/67	18/7; 20/5	27.3/26.9	2.12/2	9/13/3/NA 6/15/4/NA	25/0/0; 25/0/0	0/0
Mastroianni et al., 2022 [15]	Italy	RARC/ORC	58/58	64/66	44/14; 40/18	26/26	2.19/2.07	11/45/2/0; 12/44/2/0	NA/46/0; NA/42/NA	79/72
Khan et al., 2020 [21]	UK	RARC/ORC	20/20	68/68	17/3; 18/2	27.50/26.99	1.85/1.85	11/3/6/0; 14/0/4/2	18/2/0; 17/3/0	10/15
Venkatramani et al., 2020 [22]	USA	RARC/ORC	150/152	70/67	126/24; 128/24	27.8/28.2	NA	48/82/6/4; 51/81/16/4	113/36/1; 122/30/0	24/20
Bochner et al., 2018 [23]	USA	RARC/ORC	60/58	66/65	51/9; 42/16	27.9/29	2.73/2.84	35/8/12/5; 32/7/15/4	27/33/0; 23/32/3	55/55
Parekh et al., 2018 [4]	USA	RARC/ORC	150/152	70/67	126/24; 128/24	27.8/28.2	NA	48/82/6/4; 51/81/16/4	113/36/1; 122/30/0	24/20
Khan et al., 2016 [24]	UK	RARC/ORC	20/20	68.6/66.6	17/3; 18/2	27.5/27.4	1.85/1.85	11/3/6/0; 14/0/4/2	18/2/0; 17/3/0	10/15
Bochner et al., 2015 [25]	USA	RARC/ORC	60/58	66/65	51/9; 42/16	27.9/29	2.73/2.84	35/8/12/5; 32/7/15/4	27/33/0; 23/32/3	55/55
Messer et al., 2014 [26]	USA	RARC/ORC	20/20	69.5/64.5	18/2; 16/4	27.6/28.3	3.0/3.0	7/3/3/7; 12/1/2/5	19/1/0; 18/2/0	5/10
Parekh et al., 2013 [27]	USA	RARC/ORC	20/20	69.5/64.5	18/2; 16/4	27.6/28.3	3.0/3.0	7/3/3/7; 12/1/2/5	19/1/0; 18/2/0	5/10
Nix et al., 2010 [19]	USA	RARC/ORC	21/20	67.4/69.2	14/7; 17/3	27.5/28.4	2.71/2.70	6/12/3/0; 5/14/1/0	14/7/0; 14/6/0	33/30

*BMI* Body mass index, *ASA* American Society of Anesthesiologists physical status classification, *RARC* Robot-assisted radical cystectomy, *ORC* Open radical cystectomy, *NA* Not available

### Risk of bias

**Fig. 2:**
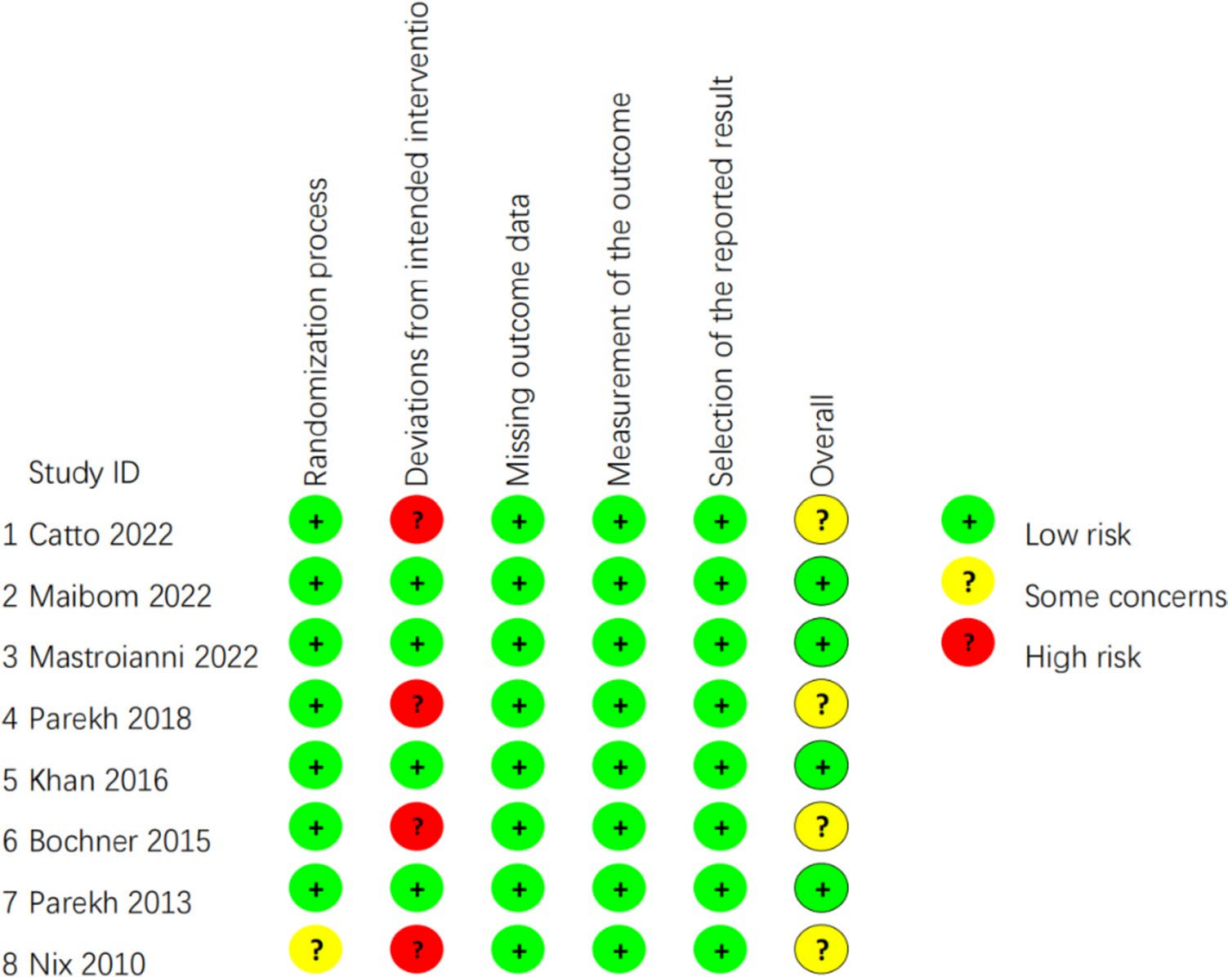
Quality assessment of each study

Each study included in the meta‑analysis was RCT. According to RoB 2 [18], the majority of the listed RCTs were categorized as “low risk of bias” or “some concerns”. The bias of quality assessment is shown in Fig. 2.

### Oncologic outcomes

The oncologic outcomes evaluated in this meta-analysis included overall survival, recurrence-free survival and recurrence patterns.

#### Overall survival

**Fig. 3:**
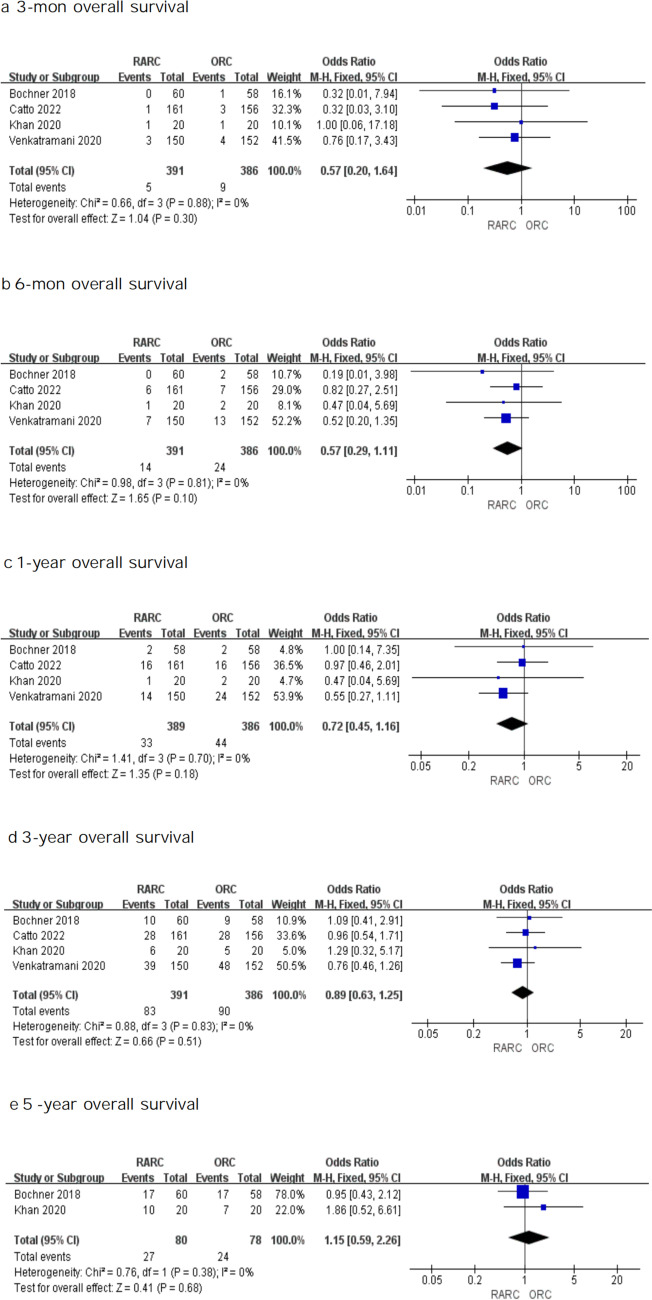
Forest plots showing (**a**) 3-month overall survival, (**b**) 6-month overall survival, (**c**) 1-year overall survival, (**d**) 3-year overall survival, and (**e**) 5-year overall survival in RARC group and ORC group. MH mantel–haenszel, CI confidence interval, df degrees of freedom

Four RCTs assessed OS between RARC and ORC. As seen in Fig. 3, the OS analysis in bladder cancer suggested no significant difference in survival rates between the two approaches at 3-mon (OR: 0.57; 95% CI 0.20 – 1.64; *P* = 0.30), 6-mon (OR: 0.57; 95% CI 0.29 – 1.11; *P* = 0.10), 1-year (OR: 0.72; 95% CI 0.45 – 1.16; *P* = 0.18), 3-year (OR: 0.89, 95% CI 0.63—1.25; *P* = 0.51) and 5-year (OR: 1.15, 95% CI 0.59—2.26; *P* = 0.68) follow-up. This result indicated that when compared with the ORC, the RARC group had equivalent effectiveness when it came to overall survival.

#### Recurrence-free survival

**Fig. 4:**
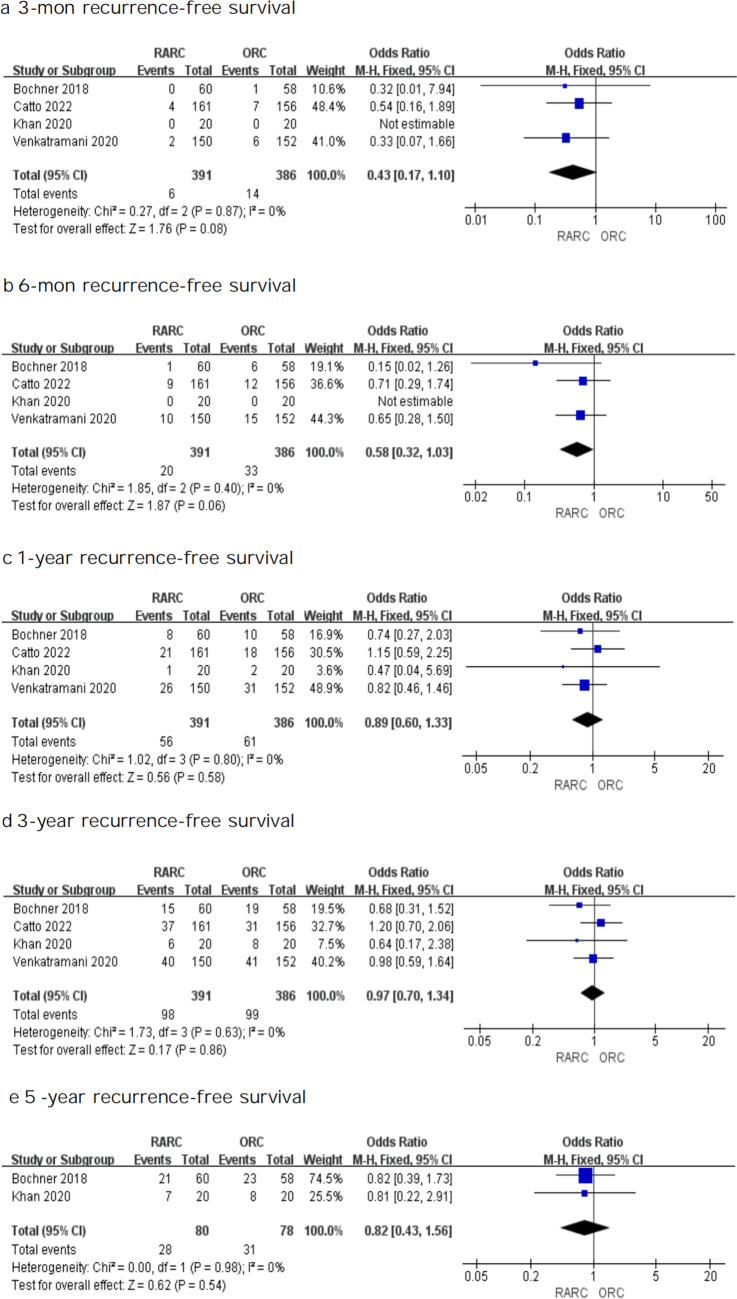
Forest plots showing (**a**) 3-month recurrence-free survival, (**b**) 6-month recurrence-free survival, (**c**) 1-year recurrence-free survival, (**d**) 3-year recurrence-free survival, and (**e**) 5-year recurrence-free survival in RARC group and ORC group. MH mantel–haenszel, CI confidence interval, df degrees of freedom

In order to determine recurrence-free survival, four RCTs were examined. There was little significant difference between the two approaches for the 3-month (OR: 0.43, 95% CI 0.17—1.10; *P* = 0.08), 6-mon (OR: 0.58, 95% CI 0.32—1.03; *P* = 0.06), 1-year (OR: 0.89, 95% CI 0.60—1.33; *P* = 0.58), 3-year (OR: 0.97, 95% CI 0.70—1.34; *P* = 0.86) and 5-year (OR: 0.82, 95% CI 0.43—1.56; *P* = 0.54) recurrence-free survival as shown in Fig. 4, which indicated that individuals who experienced RARC or ORC had similar recurrence-free survival.

#### Recurrence patterns

**Fig. 5:**
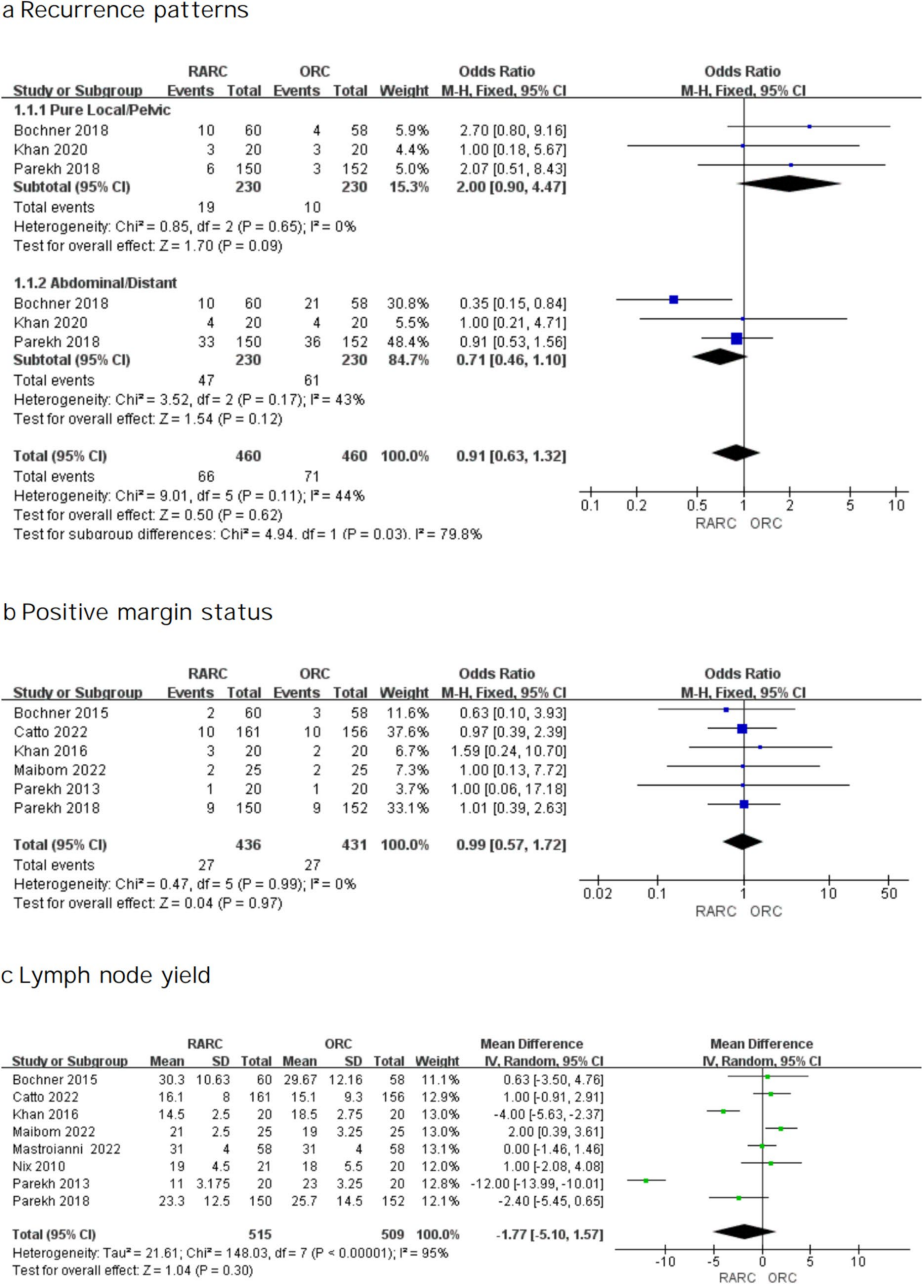
Forest plots showing (**a**) recurrence patterns, (**b**) positive margin status, and (**c**) lymph node yield

Three RCTs were analyzed in terms of reported local (pelvic) and abdominal/distant recurrence data. Among the studies, no high risk of heterogeneity was found, so a fixed effects model was selected. As shown in Fig. 5a, we found neither RARC nor ORC was related to a substantially greater chance of a local or abdominal/distant recurrence. Nevertheless, regarding recurrence patterns, a significant difference was found between the two approaches (test for subgroup differences, *P* = 0.03).

### Pathological outcomes

Six investigations including 867 participants reported PSM rates. The heterogeneity test suggested I^2^ = 0%, so we used a fixed effect model. No difference in PSM was found (OR: 0.99; 95% CI 0.57—1.72; *P* = 0.97 Fig. 5b). Likewise, no significant difference was found in terms of lymph node yield (WMD: -1.77; 95% CI -5.10 -1.57; *P* = 0.30 Fig. 5c).

### Perioperative outcomes

#### Intraoperative outcomes: operative time, EBL and blood transfusion rate

##### Operative time

**Fig. 6:**
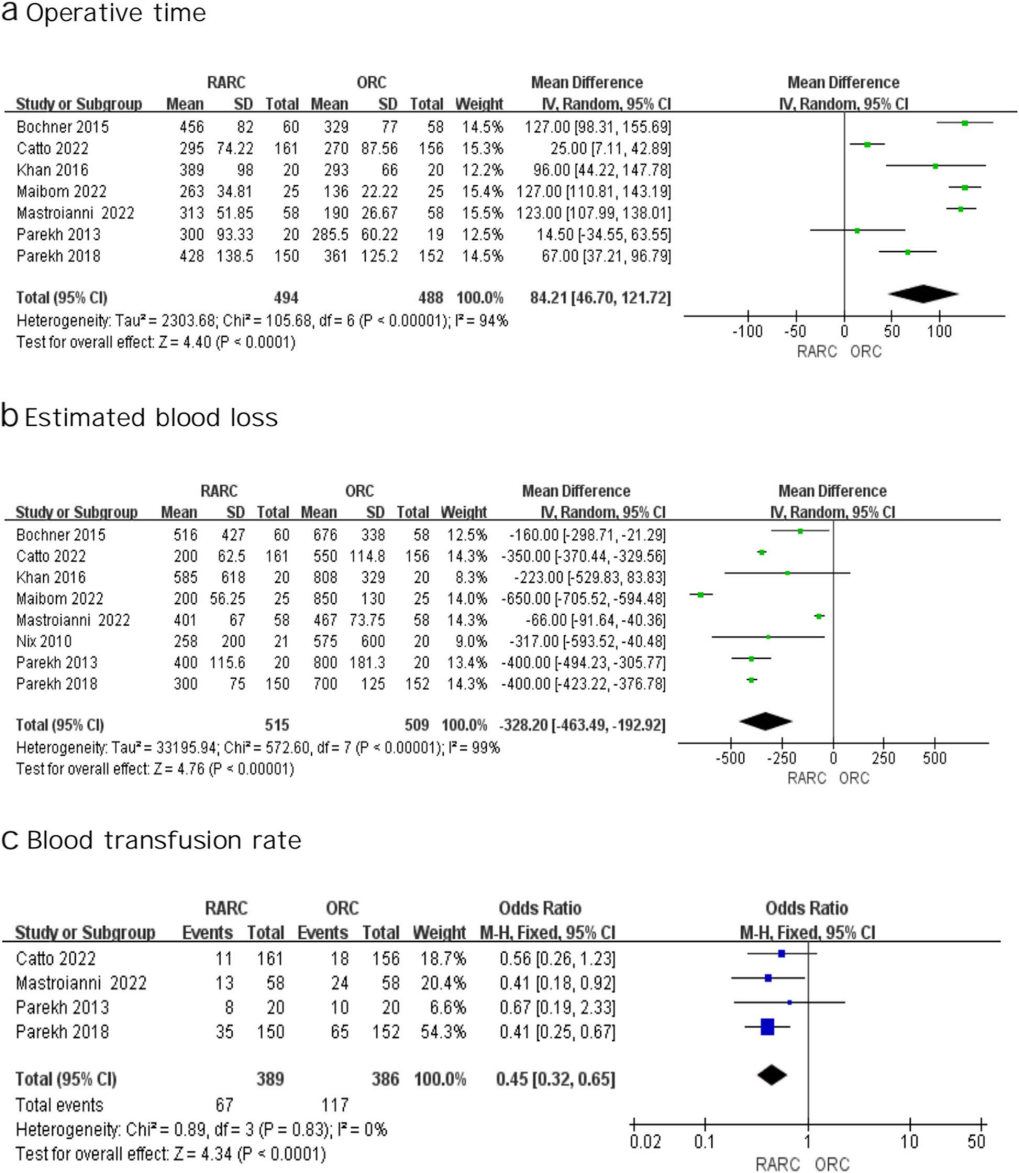
Forest plots showing (**a**) operative time, (**b**) estimated blood loss, and (**c**) blood transfusion rate

High heterogeneity was identified between studies (I^2^ = 94%), so we conducted the analysis using a random effect model. As shown in Fig. 6a, the forest plots indicated that RARC was related to longer operative time (WMD: 84.21; 95% CI 46.70 -121.72; *p* < 0.0001 Fig. 6a).

##### Estimated blood loss

Eight investigations showed the EBL [4, 14–16, 19, 24, 25, 27]. A random effect model was applied according to the findings of the heterogeneity test (I^2^ = 99%). Data from 1024 patients revealed that the EBL of RARC group was much lower than that of the ORC group. (WMD: -328.2; 95% CI -463.49—-192.92; *p* < 0.00001 Fig. 6b).

##### Blood transfusion rate

No high risk of heterogeneity was found, so we used a fixed effects model. Blood transfusion rates were found to be lower in RARC (OR: 0.45; 95% CI 0.32 – 0.65; *p* < 0.0001 Fig. 6c).

#### Analysis of postoperative complications

**Fig. 7:**
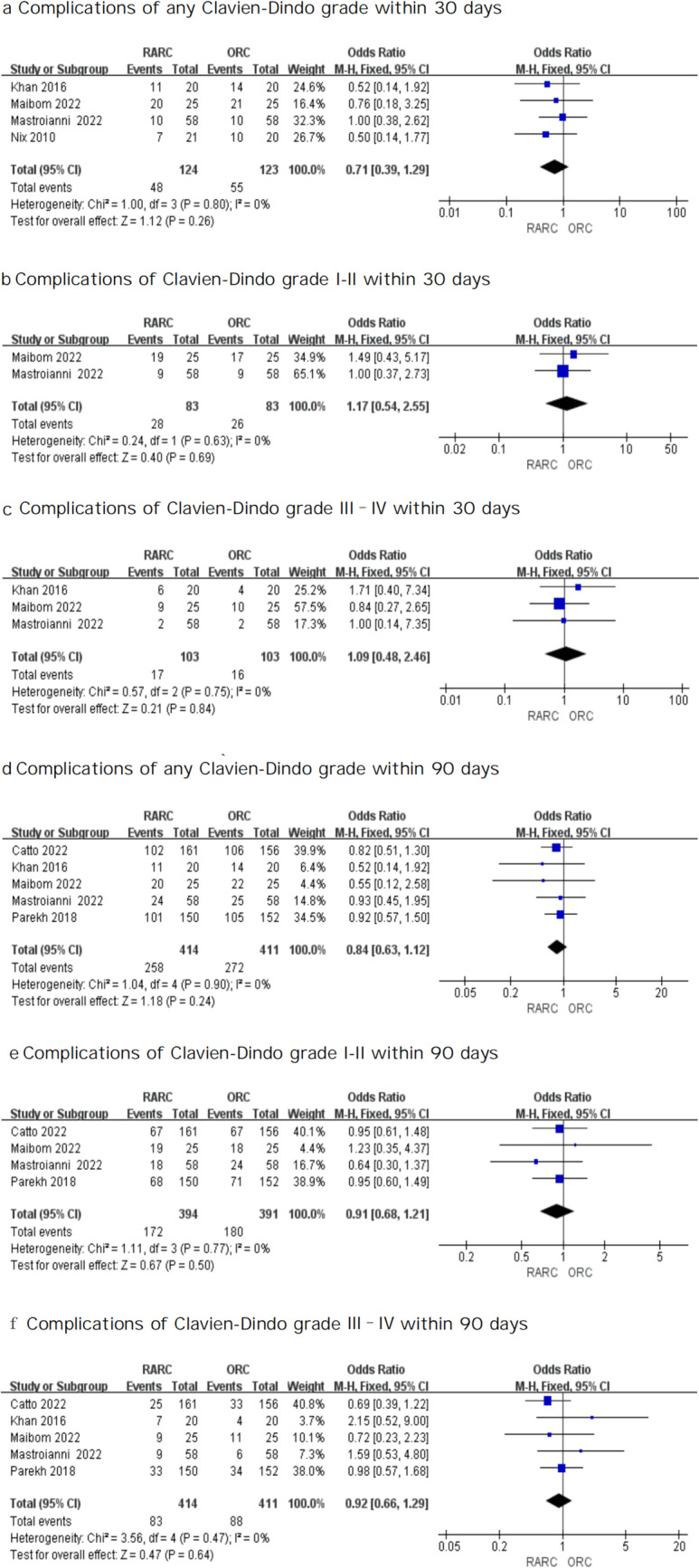
Forest plots showing (**a**) complications of any Clavien-Dindo grade within 30 days, (**b**) complications of Clavien-Dindo grade I-II within 30 days, (**c**) complications of Clavien-Dindo grade III–IV within 30 days, (**d**) complications of any Clavien-Dindo grade within 90 days, (**e**) complications of Clavien-Dindo grade I-II within 90 days, and (**f**) complications of Clavien-Dindo grade III–IV within 90 days

We evaluated postoperative complications within 30 days and 90 days following surgery. As for the postoperative complication grades of RARC and ORC within 30 days after surgery, the analysis did not identify any significant difference: any Clavien–Dindo grade (OR: 0.71; 95% CI 0.39 – 1.29; *P* = 0.26 Fig. 7a), Clavien–Dindo grade I–II (OR: 1.17; 95% CI 0.54 – 2.55; *P* = 0.69 Fig. 7b), Clavien–Dindo grade III–IV (OR: 1.09; 95% CI 0.48 – 2.46; *P* = 0.84 Fig. 7c). Likewise, we observed no significant difference regarding postoperative complication within 90 days between both groups: any Clavien–Dindo grade (OR: 0.84; 95% CI 0.63 – 1.12; *P* = 0.24 Fig. 7d), Clavien–Dindo grade I–II (OR: 0.91; 95% CI 0.68 – 1.21; *P* = 0.50 Fig. 7e), Clavien–Dindo grade III–IV (OR: 0.92; 95% CI 0.66 –1.29; *P* = 0.64 Fig. 7f).

### Length of stay

**Fig. 8:**
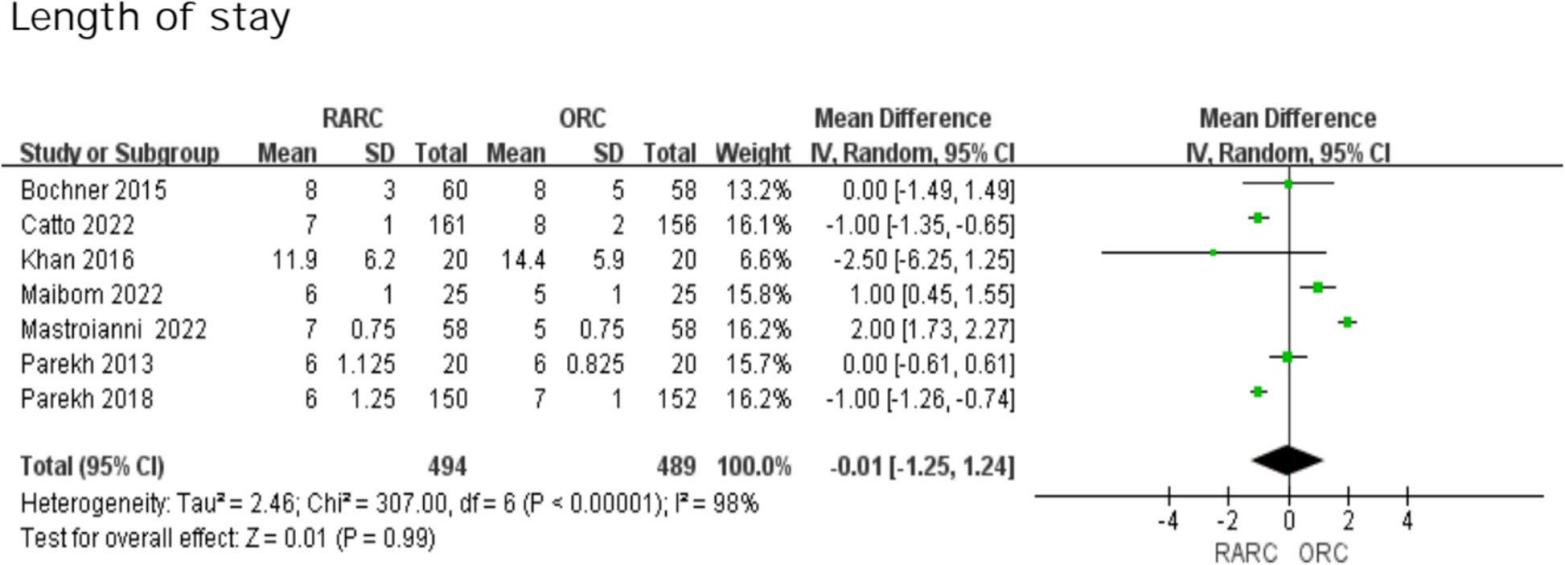
Forest plots showing Length of stay

We adopted a random effects model because of high heterogeneity (I^2^ = 98%). Evaluation of combined data from seven trials found no significant difference in LOS between the two groups (WMD: -0.01; 95% CI -1.25—1.24; *P* = 0.99 Fig. 8).

### Postoperative health-related QOL outcomes

Although six RCTs reported postoperative health-related QOL (Table 2), different quality-of-life assessment tools were used. As a result, it might not be possible to conduct a pooled analysis of data. The FACT-VCI was adopted by Messer et al. [26] to assess changes in health-related QOL scores between the two approaches at baseline, 3, 6, 9 and 12 months following surgery in 40 patients. In order to compare health-related QOL at baseline, 3 and 6 months after surgery, Bochner et al. [25]and Parekh et al. [4] adopted the QLQ-C30 and FACT-VCI, respectively, while after a mean of 8 months after surgery, Khan et al. [24] completed the FACT-BI. Vejlgaard et al. [20] evaluated health-related QOL at baseline and 3 months after surgery using QLQ-C30. According to all research, health-related QOL of the two groups did not significantly differ from one another. Nevertheless, in a new trial, Catto et al. [14] used several measures in 317 participants to evaluate health-related QOL and concluded those who underwent robotic surgery showed better QOL vs open surgery at 5 weeks (difference in EQ-5D-5L scores, –0.07 [95% CI, –0.11 to –0.03]; *P* = 0.003) and less disability at 5 weeks (WHODAS 2.0 scores, 0.48 [95% CI, 0.15–0.73]; *P* = 0.003) and at 12 weeks (difference in WHODAS 2.0 scores, 0.38 [95% CI, 0.09–0.68]; *P* = 0.01). By 26 weeks, there was little difference in terms of QOL and disability scores.

**Table 2:** Postoperative health-related QOL change

Author	QOL scale	Arm	QOL score baseline	QOL score at 1 month	QOL score at 3 months	QOL score at 6 months	QOL score at 9 months	QOL score at 12 months	QOL score overall
Catto 2022 [14]	EQ-5D-5L	RARC/ORC	0.89/0.90	0.83/0.77 (*P* = 0.003)	0.88/0.86 (*P* = 0.1)	0.87/0.87 (*P* = 0.2)	NR	NR	NR
	QLQ-C30		87.55/87.74	76.46/68.66 (*P* < 0.001)	86.06/81.96 (*P* = 0.01)	87.14/84.86 (*P* = 0.17)	NR	NR	NR
	QLQ-BLM30		NR	NR	NR	NR	NR	NR	NR
	WHODAS-2		9.3/9.2	20.9/26.4 (*P* = 0.003)	10.2/14.9 (*P* = 0.01)	10.8/11.1 (*P* = 0.24)	NR	NR	NR
Vejlgaard 2022 [20]	QLQ-C30	RARC/ORC	77/78	NR	84/77 (*P* = 0.47)	NR	NR	NR	NR
Parekh 2018 [4]	FACT-VCI	RARC/ORC	120.1/120.9	NR	122.8/125.2	126.0/127.5	NR	NR	NR
Khan 2016 [24]	FACT-Bl	RARC/ORC	NR	NR	NR	NR	NR	NR	NR
Bochner 2015 [25]	QLQ-C30	RARC/ORC	78/75	NR	77/72 (*P* = 0.40)	76/78 (*P* = 0.5)	NR	NR	NR
Messer 2014 [26]	FACT-VCI	RARC/ORC	119/135	NR	126.5/135.5 (*P* = 0.85)	121.5/126 (*P* = 0.58)	141.5/127.5 (*P* = 0.63)	116/129 (*P* = 0.48)	NR

*QOL* Quality of life, *RARC* Robot-assisted radical cystectomy, *ORC* Open radical cystectomy, *NA* Not available

## Discussion

Bladder cancer poses significant cumulative morbidity because of the advanced age and substantial smoking prevalence. Due to the intricacy of the procedure and the patients' intrinsic weakness, radical cystectomy—the recommended therapy for patients with very high-risk and muscle-invasive bladder cancer—is related to a significant prevalence of complications [1, 28]. Despite their greater cost and steeper learning curve, minimally invasive operations like RARC are being used in many areas of medicine [28, 29]. RARC has gained popularity in bladder cancer treatment because of its prospective benefits. According to reports, the percentage of cystectomies carried out with the RARC increased from 0.6% to 12.8% [30]. With a focus on the differences in results between RCTs, the purpose of the updated meta-analysis was to compare the most recent evidence on the differential influence of these two strategies on oncologic, perioperative, and health-related (QOL) outcomes as well as complication-related outcomes.

Here we report the largest RCT outcomes analysis between RARC and ORC, including 1024 patients from eight studies. As a malignant tumor, bladder cancer patients' long-term follow-up oncological outcomes are a major concern for surgeons. Our analysis of the existing literature showed equivalent oncologic results. Besides, we didn't observe a difference in recurrence patterns, OS or RFS in all RCTs, indicating that RARC is a safe procedure, which has long-term survival effectiveness for bladder cancer that is comparable to that of ORC.

These conclusions are based on hypothesis and could be accessed further in a meta-analysis of individual patient data according to properly considered and standard definitions of recurrence locations. PSM and lymph node yield have a significant effect on postoperative survival. We also came to a conclusion from our study that there were no significant differences in PSM and lymph node yield.

Consistent with the benefits of the robotic approach for other malignant neoplasms, we found that RARC was related to lower blood loss and lower transfusion rates at the expense of prolonged operative time in terms of perioperative safety [31]. RARC has significant advantages in controlling bleeding, which may be due to clearer operational vision, more elaborate manipulation and the use of hemostatic devices in robotic-assisted surgery. In addition, another major factor affecting bleeding is the pneumoperitoneum used in robot-assisted surgery. The increase of pressure is conducive to the occlusion of small blood vessels, reducing the amount of bleeding [32].

In terms of the complication rate within 30 and 90 days, we observed no difference between ORC and RARC. Fewer postoperative complications are a potential benefits of RARC [33–36]. Yet, the RARC's benefit may not always be apparent. Additionally, in the Clavien–Dindo subgroup analyses, no significance was found between the two groups regarding minor and major complications at 30 days and 90 days following surgery. Urinary tract infections are typical complications following radical cystectomy [37]. Urinary diversions, however, make it hard to prevent these consequences. Despite continual improvements in surgical methods, similar complications have happened on occasion. Thus, there remains a great need to study postoperative complications [38].

Urinary construction is a key factor in postoperative complications, which is a controversial subject, with intracorporeal and extracorporeal options to choose from. In this meta-analysis, there are only three RCTs performed ICUD method [14–16]. Hussein et al. [9] analyzed 2,125 patients with radical cystectomy and concluded that the postoperative complications of ICUD decreased over time. However, ICUD has intrinsic difficulties, such as a challenging learning curve, insufficient clinical experience of the surgeons, unreasonable operating times, and the possibility of undermining the quality of the uretero-ileal anastomoses outweighing its advantages [39, 40]. Further studies about the procedure of urinary construction are needed, and it is essential to improve the understanding and management of these serious and common adverse reactions.

HRQOL relates to the influence of illness and therapy on one's physical, mental, and social spheres as related to overall well-being [41], which is considered to be one of the important parameters after malignant tumor treatment [42, 43]. As several techniques were used to assess QOL, it was difficult to pool QOL data for our study. Five RCTs found no significant difference regarding health-related QOL. However, a new study comparing recovery after RARC with intracorporeal reconstruction vs ORC suggested that any statistically significant change at five weeks was in favour of robotic surgery in terms of postoperative health-related QOL outcomes [14]. This might be due to the change in urinary reconstruction and the large sample size in this study.

In this review, we also observed no significant difference in LOS, which reflect similar complication rates between the two procedures.

Compared with some existing meta-analyses [44–46], this study conducted the most comprehensive analysis of RCTs, both in terms of the number of RCTs included and the indicators analyzed. In addition to analyzing indicators such as PSM, EBL, blood transfer rates, ORT, LOS, etc. We also analyzed lymph node yield and health related QOL. We used more detailed methods to analyze survival indicators and postoperative complications, and conducted subgroup analysis of recurrence patterns. Nevertheless, several limitations cannot be avoided. First, a limited sample size of just 8 randomized controlled trials (1024 patients) raises concerns about the validity of our findings on effectiveness and safety. Next, the majority of the studies were not considered to use a blinding procedure for their participants, and thus there may be potential bias. The lack of information on postoperative complications is a disadvantage. Future research should follow criteria for evaluating and reporting of postoperative complications. Health-related QOL assessment tools differ significantly between eligible RCTs. In addition, most studies were performed at a single center, which is reflected in the results of the heterogeneity test of operative time and might reflect the experience of the individual surgeon. Furthermore, some included studies were carried out with enhanced recovery after surgery (ERAS) programmes. ERAS is a standardized, multimodal, and multidisciplinary scientific concept for perioperative management. ERAS aims to decrease intra-operative blood loss, decrease postoperative complications, and reduce recovery times. The main content of ERAS in urology includes admission assessment, preoperative preparation, intraoperative measures, and postoperative management. In particular, bleeding can be reduced by perioperative fluid management. Additionally, restrictive intraoperative hydration, along with norepinephrine administration, decreases intraoperative blood loss [47]. According to a study from the University of Sheffield [48], the use of the ERAS setting was related to less blood loss and quicker recovery time following radical cystectomy, which related to study heterogeneity. It should be noted our single center has been implementing ERAS since 2015, and a retrospective cohort study including 192 patients from our center shows that ERAS may successfully speed up patient rehabilitation and is associated with less blood loss and LOS [49]. Furthermore, we performed a meta-analysis about this theme, evaluating and confirming the effectiveness of ERAS in the perioperative outcomes of radical cystectomy[50].

## Conclusion

In conclusion, RARC had similar oncological outcomes compared with ORC as shown in our systematic review and meta-analysis. RARC leads to less EBL and lower blood transfusion rates at the expense of prolonged operative time. RARC and ORC resulted in similar rates of postoperative complications and health-related QOL. Further well-designed RCTs are essential to confirm this conclusion.

## Data Availability

The datasets that support the findings of this study are available from the corresponding author on reasonable request.

## Abbreviations

ROB2: The version 2 of the Cochrane risk-of-bias tool for randomized trials
RARC: Robot‐assisted radical cystectomy
ORC: Open radical cystectomy
QOL: Quality of life
ICUD: Intracorporeal urinary diversion
RCTs: Randomized controlled trials
PRISMA: Preferred Reporting Items for Systematic Reviews and Meta-Analyses
OS: Overall survival
RFS: Recurrence-free survival
PSM: Positive margin status
EBL: Estimated blood loss
LOS: Length of stay
WMD: Weighted mean difference
CI: Confidence intervals
OR: Odds ratio
FACT-VCI: Functional Assessment of Cancer Therapy-Vanderbilt Cystectomy Index
QLQ-C30: Quality of Life Questionnaire Core 30
FACT-BI: the Functional Assessment of Cancer Therapy – Bladder
EQ-5D-5L: The EuroQol-5 dimension-5 level
WHODAS 2.0: WHO Disability Assessment Schedule 2.0
ERAS: Enhanced recovery after surgery
ASA: American Society of Anesthesiologists physical status classification
BMI: Body mass index
